# Nimotuzumab Increases the Recovery Rate of Severe and Critical COVID-19 Patients: Evaluation in the Real-World Scenario

**DOI:** 10.3389/fpubh.2022.948520

**Published:** 2022-07-22

**Authors:** Henrry Diaz, Jorge Jiménez, Aray Hernández, Leivis Valdés, Ariadna Martínez, Leonor Porto, Raity Hernández, Nadina Travieso, Julio Héctor Jova, Loipa Medel, Mayelin Troche, Annia Gorte, Delmis Batista, Ana Rosa Valls, Leticia Cabrera, Milagros Domeq, Leslie Pérez, Patricia Lorenzo-Luaces, Lizet Sánchez, Danay Saavedra, Mayra Ramos, Tania Crombet

**Affiliations:** ^1^Julio Trigo Hospital, Havana, Cuba; ^2^Salvador Allende Hospital, Havana, Cuba; ^3^León Cuervo Rubio Hospital, Pinar del Río, Cuba; ^4^10 de Octubre Hospital, Havana, Cuba; ^5^Amalia Simone Hospital, Camagüey, Cuba; ^6^Mario Muñoz Hospital, Matanzas, Cuba; ^7^Joaquín Castillo Hospital, Santiago de Cuba, Cuba; ^8^Gustavo Aldereguía Hospital, Cienfuegos, Cuba; ^9^Center of Molecular Immunology, Havana, Cuba

**Keywords:** brief research report nimotuzumab, COVID-19, SARS-CoV-2, inflammation, fibrosis, EGFR, monoclonal antibody

## Abstract

EGFR signaling is an important regulator of SARS-CoV induced lung damage, inflammation and fibrosis. Nimotuzumab is a humanized anti-EGFR antibody registered for several cancer indications. An expanded access study was conducted to evaluate the safety and recovery rate of severe and critical patients with confirmed SARS-CoV-2 infection, treated with nimotuzumab in combination with the standard of care in the real-world scenario. The antibody was administered as an intravenous infusions every 72 h, up to 5 doses. In order to assess the impact of nimotuzumab, the recovery rate was compared with a paired retrospective cohort. Control patients received standard treatment according the national protocol but not nimotuzumab. Overall, 1,151 severe or critical patients received nimotuzumab in 21 hospitals of Cuba. Median age was 65 and 773 patients had at least one comorbidity. Nimotuzumab was very well-tolerated and mild or moderate adverse events were detected in 19 patients. 1,009 controls matching with the nimotuzumab patients, were selected using a “propensity score” method. The 14-day recovery rate of the nimotuzumab cohort was 72 vs. 42% in the control group. Controls had a higher mortality risk (RR 2.08, 95% CI: 1.79, 2.38) than the nimotuzumab treated patients. The attributable fraction was 0.52 (95% CI: 0.44%; 0.58), and indicates the proportion of deaths that were prevented with nimotuzumab. Our preliminary results suggest that nimotuzumab is a safe antibody that can reduce the mortality of severe and critical COVID-19 patients.

## Introduction

Growth factor receptors can be altered after a viral infection (1). Remarkably, the overexpression of some receptors may promote viral replication and immune response evasion (1). Representative altered receptor pathways include fibroblast growth factor (FGF), transforming growth factor beta (TGF beta), and epidermal growth factor receptor (EGFR) (2). Particularly, upon a viral infection, EGFR overactivation could have a major role in the inflammatory response and mucus production (1, 3). According to Hirano et al., EGFR participates in a positive feedback loop that enhances the inflammatory responses by preventing the inhibition through the negative regulator SOCS3 (suppressor of cytokine signaling-3) (4). Furthermore, a crucial regulatory role of the EGFR in thrombin-mediated inflammation was recently reported (4, 5).

EGFR can also be overexpressed after a lung injury (6). Alveolar type II cells in fibrotic lung tissues may express high levels of EGFR resulting in hyperplasia of the alveolar epithelial cells (4, 5). Preclinical studies of SARS-CoV-1 infections indicated that EGFR is upregulated and its overexpression contributes to enhanced lung disease (2). Particularly, in SARS-CoV-2 infected lungs, EGFR would be overexpressed after the acute lung injury or by the reduced Signal Transducer and Activator of Transcription 1 (STAT1) activity (7).

The blockade of EGFR emerges as a novel strategy for COVID-19 patients, provided its role in inflammation, immune-thrombosis and fibrosis (8, 9). Nimotuzumab is a humanized anti-EGFR monoclonal antibody, which is approved for the treatment of several epithelial tumors (10–14). The antibody efficiently inhibits the EGFR associated signal transduction, prevents proliferation, arrests the cell cycle in the G1 phase and decreases interleukin 6 (IL-6) secretion by the cancer cells (15, 16).

A phase I clinical trial evaluating nimotuzumab was conducted in moderate and severe COVID-19 patients. Forty-one patients were included in the trial. Seven patients received one dose of nimotuzumab, 29 received 2 infusions while 5 subjects required 3 doses. The antibody was very safe. Recovery rate was 80.64% in severe patients. Inflammatory markers decreased overtime and interleukin-6 concentration diminished from 46.5–14.51 pg/ml at day 7 (9).

Then, an expanded access study was launched nation-wide to evaluate the safety and recovery rate of severe or critical COVID-19 patients in comparison with matched controls in the real-world scenario.

## Materials and Methods

An expanded access study was conducted to evaluate the safety and recovery rate of patients with confirmed SARS-CoV-2 infection by reverse transcription polymerase chain reaction (RT-PCR), in the conditions of the standard medical practice.

Subjects older than 18 of any gender or skin color with severe or critical illness were enrolled. Patients with severe disease were those individuals who have oxygen saturation (SpO2) <94% on room air at sea level, a ratio of arterial partial pressure of oxygen to fraction of inspired oxygen (PaO2/FiO2) <300 mm Hg, a respiratory rate of 30 breaths/min or more, or lung infiltrates >50% while subjects with critical illness were defined as those with respiratory failure, septic shock, and/or multiple organ dysfunction (17). In addition, patients received other drugs included in the national protocol for COVID-19, including steroids, anticoagulants and antibiotics. Patients did not receive any other immunoregulatory compound and were not vaccinated at the moment of enrollment. At the moment of the retrospective study (July–October 2021), the delta variant of concern was the dominant strain nation-wide (18).

Nimotuzumab was administered as an intravenous (IV) infusions every 72 h, up to 5 doses. The recommended number of doses was 3. The loading dose was 200 mg, followed by 100 mg in the next infusions. The antibody was diluted in 250 mL of saline solution (0.9%), administered over 2 h.

In order to assess the impact of nimotuzumab in the recovery rate, information from patients with severe or critical disease was collected from the national database of the Cuban Ministry of Health (9,027 patients). Control patients received standard treatment according the state COVID-19 guideline but not nimotuzumab or any other immunomodulatory drug. In order to guarantee that both cohorts were balanced, controls matching with the nimotuzumab patients were selected with a “propensity score” method, using demographics, province of residence as well as the most important comorbidities associated with COVID-19 prognosis. Briefly, a logistic regression score was assigned to each patient, considering the nimotuzumab treatment variable as the dependent variable and age, sex, comorbidities and province of residence as independent. Overall, 1,009 patients not treated with nimotuzumab or immunomodulatory drugs were selected with an exactly coincident score (0 tolerance), as compared to the patients treated with the antibody. The homogeneity of the groups was verified using the Pearson's chi-square and the *t*-student tests. The recovery rate from COVID-19 was estimated for treated and control patients at 14 days. Then, the relative risks of death from COVID-19 between the studied cohorts were estimated by using chi-square association test. The nimotuzumab and population attributable fractions, representing the number of deaths that could be prevented with nimotuzumab administration, were also estimated. Finally, a logistic regression analysis was performed in each subgroup defined by the control variables to estimate the mortality risk ratio, i.e., the relative increase in the probability of death rather than recovery of patients not receiving nimotuzumab. The risk ratio estimates and their 95% CI in each subgroup were displayed in a forest plot. Descriptive statistic was used to analyze the data set. The analyses were made with the SPSS version 25.0. The study was conducted according to the Helsinki ethical principles for medical research involving human subjects. The study was funded by the Cuban Ministry of Health and the Center of Molecular Immunology. The trial was approved by the ethical review board of the leading institutions and the protocol was listed in the public registry of clinical trials (https://rpcec.sld.cu/ensayos/RPCEC00000369-En).

## Results

From July 8 to October 27, 2021, 1,151 severe or critical patients received treatment with nimotuzumab in 21 hospitals from 9 provinces of Cuba. Median age was 65 (18–99) and 773 patients (67.2%) had at least one associated primary condition. Most common comorbidities were hypertension (57.5%), diabetes mellitus (20.8%), cardiovascular disease (14.2%), bronchial asthma (8.3%) chronic obstructive pulmonary disease (COPD) (3.9%), chronic kidney disease (0.8%) and cancer (0.2%).

Patients received nimotuzumab concomitantly with the standard of care including low molecular weight heparin, steroids and antibiotics. All patients were treated in the intensive care unit (ICU). The mean time between hospitalization and nimotuzumab was 2.7 days. Overall, 552 patients (48%) received a single infusion of the antibody, 245 subjects (21.3%) received 2 doses, 313 patients (27.2%) needed 3 nimotuzumab doses, while 28 (2.4%) and 13 patients (1.1%) required 4 or 5 antibody doses, respectively, at the discretion of the treating physicians.

Nimotuzumab was very well-tolerated and mild or moderate adverse events were detected in 19 patients (1.65%). Adverse events consisted mainly on chills, tremors, fever, headache, dyspnea and hypotension. Most adverse events were detected after the first antibody infusion. The 14-day lethality rate was 20.8%. Severe and critical patients without comorbidities had significantly less mortality as compared with those with 1 associated condition or more. The lethality rate was 15.8% in subjects without any chronic disease and 22.3% in subjects with one or more previous condition. The lethality rate decreased in relation with the number of doses. The l4-day lethality rate was 24.3%, 21.6%, 13.4%, 10.7% and 0, for patients receiving from 1 to 5 nimotuzumab doses, in that order.

To preliminary assess the impact of nimotuzumab, a paired retrospective cohort study was done. Globally, 1,009 controls matching with the nimotuzumab patients, were selected using a “propensity score” method. Control patients received standard treatment according the national COVID-19 protocol but not nimotuzumab. As a measure of the success of the balance between the cohorts achieved with the matching, the reduction obtained in the R squares (*R*^2^), the likelihood ratio statistic and the omnibus tests of the logistic regression performed before and after matching are shown in Table 1.

**Table 1:** Predictive value measures of the logistic regression model before and after matching.

	**Before matching**	**After matching**
Omnibus tests of model chi-square	1,510.890	0.000
−2 log likelihood	5,094.6	2,797.5
Cox & snell R square	0.156	0.000
Nagelkerke R square	0.298	0.000

Both groups were homogeneous in terms of demographics and number of comorbidities (Table 2). As described in other series, the most frequent comorbidities of these severe and critical patients were hypertension, diabetes, cardiovascular disease and obesity. Table 2 describes the characteristics of both cohorts.

**Table 2:** Baseline characteristics of patients treated with nimotuzumab vs. controls not receiving immunomodulatory drugs.

		**Nimotuzumab** ***N*** **=** **1,009**		**Control** ***N*** **=** **1,009**		**^**χ2**^ *p*-value**
		**Freq**	**%**	**Freq**	**%**	
Sex	F	464	46.0%	423	41.9%	0.07
	M	545	54.0%	586	58.1%	
Age	Median (SD)	64.56 ± 14.9		64.67 ± 14.8		0.87^*^
Age groups	19–29	9	0.9%	9	0.9%	1.00
	30–39	41	4.1%	41	4.1%	
	40–49	120	11.9%	120	11.9%	
	50–59	227	22.5%	227	22.5%	
	60–69	192	19%	192	19%	
	70–79	238	23.6%	238	23.6%	
	80–89	154	15.3%	154	15.3%	
	90–100	28	2.8%	28	2.8%	
Comorbidities	Hypertension	644	63.8%	644	63.8%	1.00
	Diabetes	215	21.3%	215	21.3%	1.00
	COPD	32	3.2%	32	3.2%	1.00
	Cardiovascular disease	156	15.5%	184	18.2%	0.10
	Cancer	3	0.3%	3	0.3%	1.00
	Asthma	85	8.4%	68	6.7%	0.15
	Chronic kidney Disease	5	0.5%	5	0.5%	1.00
	Obesity	118	11.7%	128	12.7%	0.50

* *Student's t-test for comparison of means*.

The 14-day recovery rate for the nimotuzumab cohort was 72 vs. 42% in the control group. Controls had higher mortality risk (RR 2.08, 95% CI: 1.79, 2.38) than the nimotuzumab treated patients (Pearson's Chi-square *p* = 0.000).

In our data set, the nimotuzumab attributable fraction was 0.52 (95% CI: 0.44; 0.58), and represents the proportion of deaths that was prevented with the antibody. The population attributable fraction, which is a measure of the potential impact that nimotuzumab would have on the recovery of severe or critical patients, was also estimated. The population attributable fraction was 0.26 (95% CI: 0.22%; 0.29), and indicates that in a prospective scenario, 26% of the deaths of severe and critical patients would be avoided with nimotuzumab administration.

**Figure 1:**
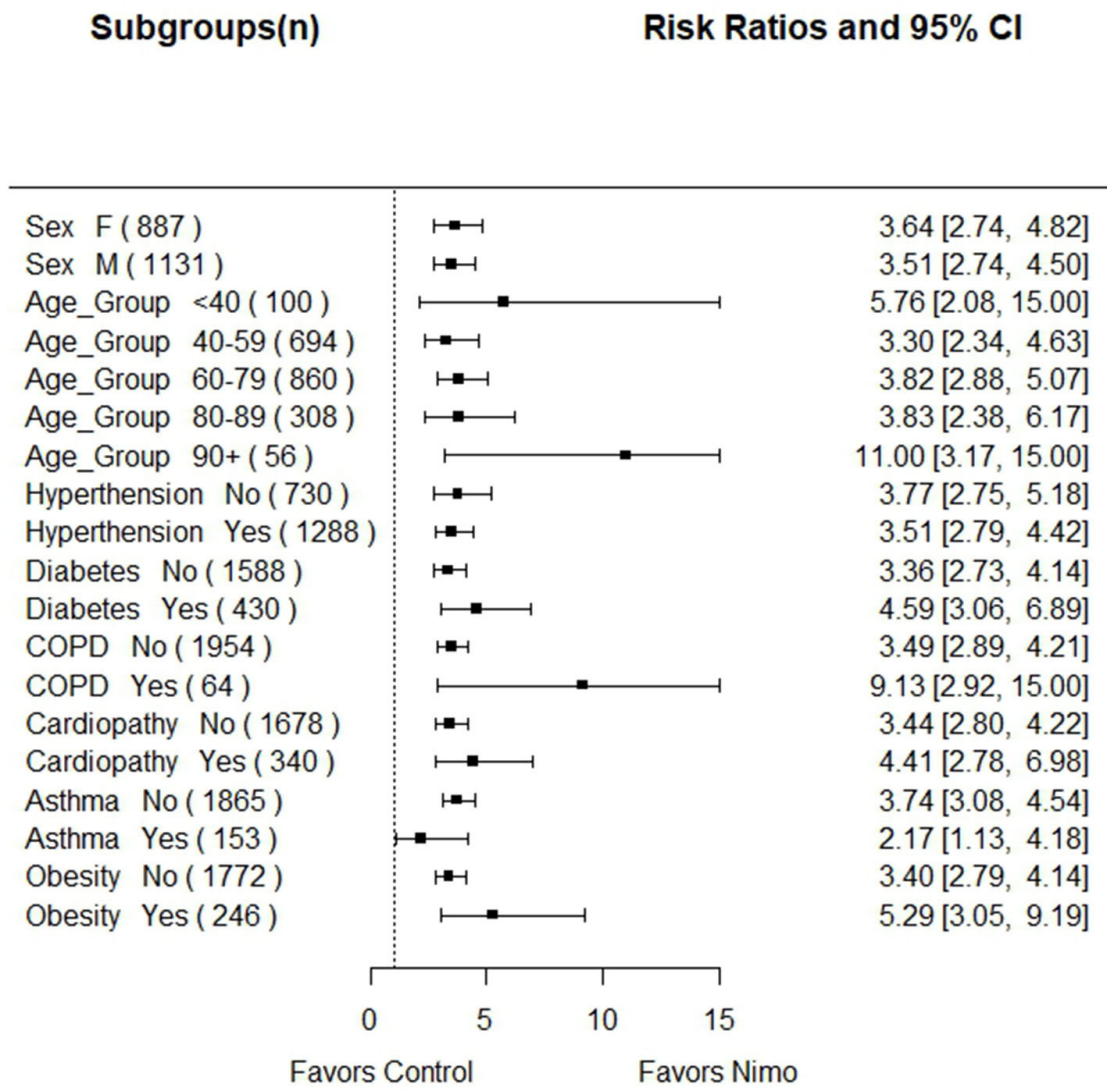
Forest plot showing the mortality risk of the control vs. nimotuzumab treated patients according demographics and comorbidities. In all subgroups, the probability of death was significantly higher in non-nimotuzumab treated subjects.

A subgroup analysis of the mortality risk of the control vs. nimotuzumab treated patients was done. The forest plot is shown in Figure 1. In all subgroups, the probability of death was significantly higher in non-nimotuzumab treated subjects. The largest treatment benefit was seen in patients older than 90 and in patients with COPD. For the subgroup of subjects older than 90, the mortality risk was 11 times higher in the control vs. nimotuzumab patients and in patients with COPD, the risk of death was 9 times higher in the control vs. nimotuzumab group.

## Discussion

EGFR is implicated in inflammation through NF-kB, angiogenesis and profibrotic events (19). Multiple pieces of evidences support the role of the EGFR in the COVID-19 pathogeny (1, 7, 20, 21). Martinez et al., found higher levels of EGFR in COVID-19 individuals vs. community associated pneumonia subjects (19) and osimertinib, a well-known EGFR antagonist, showed *in vitro* anti-SARS-CoV-2 action (22) and prevented the virus cytopathic effect (23). In addition, several phosphoproteomic studies of SARS-CoV-2- infected cells disclosed that the virus activates EGFR (24). According Camara and Brandao, EGFR is the main influential receptor involved in COVID-19 (25). In spite of the multiple theoretical and *in vitro* evidences of the key role of the EGFR in COVID-19, this is the first proof of concept that blocking EGFR can have a positive impact in decreasing COVID-19 mortality. EGFR is a very well-validated target in oncology (26) but not in COVID-19. Moreover, the use of EGFR inhibitors in the setting of COVID-19 can be controversial, due to the previous reports of interstitial lung disease in patients with lung adenocarcinoma treated with EGFR tyrosine kinase inhibitors (27). The first clinical trial in hospitalized COVID-19 patients, demonstrated that nimotuzumab was very safe and the 14-day recovery rate was 82.9% (9). Only 8 patients (19.5%) of 41 required invasive mechanical ventilation. After 7 days, 76.2% of the subjects with a severe condition, improved the PO2/FiO2 ratio and there was a significant reduction of the affected lung areas. Inflammatory markers including C-reactive protein, ferritin, lactate dehydrogenase (LDH), neutrophil to lymphocyte ratio (NLR), D-dimer, interleukin 6 and plasminogen activator inhibitor-1 (PAI-1) decreased over time (9). This manuscript reports for the first time the safety and recovery rate of patients treated with an anti-EGFR drug plus the standard of care vs. the standard of care alone, in a relatively large population in the conditions of the usual medical practice. Apart from other EGFR antibodies or small tyrosine kinase inhibitors, nimotuzumab exhibit an intermediate affinity against its target (10^−9^ M) (28). Previously, several authors have demonstrated that nimotuzumab requires bivalent binding for stable attachment to the cellular surface, leading to selectively targeting cells with high EGFR expression (29, 30). The biodistribution study in patients with epithelial tumors found that the percent of the injected dose of nimotuzumab per gram of tissue decreased 24 h post-treatment for normal organs, while the uptake in the tumor remained relatively constant (31). As a result, according its intrinsic properties, nimotuzumab would only recognize tissues with an aberrant EGFR overexpression like tumors or respiratory cells affected by infections leading to diffuse alveolar degeneration like in COVID-19 (32).

In our data set, the antibody was very safe. These results are compatible with previous findings in cancer, and represent a large advantage as compared to other biologics (11, 15, 28). Anti-inflammatory drugs, including steroids and anti-IL-6 receptor (IL-6R) or anti-TNFα antibodies can reduce tissue harm but augment the risk of sepsis (33–35). The majority of the patients required from 1 to 3 nimotuzumab doses and there was an association between the number of doses and the recovery rate. The optimal number of nimotuzumab doses needs further studies.

Under multiple circumstances, conducting traditional randomized clinical trials is not feasible, and real-world data and real-world evidence play a crucial role in taking the best medical decisions (36, 37). High-quality observational studies provide a useful platform for all clinical evaluations (38). Its greatest limitations consist of the biases induced by possible confounding factors (39, 40). The propensity score, defined as the conditional probability of receiving treatment given the covariates, can be used to balance the covariates and thus, reduce bias (41, 42). To estimate the propensity score, the distribution of the treatment variable is modeled given the observed covariates (41, 42).

In the present study, information was collected from all severe or critical patients (1,151) treated with nimotuzumab in 21 hospitals from July to October 2021. Then, patients were matched with other severe or critical COVID-19 subjects from the Cuban national database (9,027 cases) not receiving nimotuzumab or any other immunomodulatory drug. The propensity score included age, sex, eight different comorbidities, the province of residence as well as the treatment period as independent variables. After applying the propensity score method, the sample size obtained in each cohort (1,009 patients), granted a statistical power of 100% to detect differences in proportions, using the Mantel-Haenszel Chi-Square Test, at a significance level of 0.01.

Although this was not a randomized study, our preliminary data suggest that nimotuzumab reduced the relative risk of death in comparison to the matched controls. The 14-day recovery rate of the nimotuzumab cohort was 30% higher than the control group (RR 72 vs. 42%), and controls had twice the mortality risk than the nimotuzumab treated patients. The observational study concluded that 52% of the deaths among the ICU patients were prevented with nimotuzumab treatment. All patient subgroups benefitted from therapy and particularly, nimotuzumab seems to be very efficacious for vulnerable patients like those older than 90 or with COPD associated comorbidity.

Other drugs devoted to decrease the hyperinflammatory response have been evaluated. For patients treated at the intensive care units (severe or critical), the addition of one or two doses of tocilizumab, an anti-IL6R antibody, to usual care significantly reduced the mortality rate compared with usual care alone (31 vs. 35%) (relative risk 0.85; 95% CI, 0.76–0.94) (33). In another controlled trial in 3,924 severe or critical patients, 40.6% of patients who did not receive therapy with the anti-IL-6R antibody died while the mortality rate was 28.9% after the use of tocilizumab, respectively (43). The case fatality rate of severe and critical patients treated with nimotuzumab plus the standard of care (28%) compares very favorably with the rates reached after tocilizumab.

In summary, our results suggest that nimotuzumab is a safe antibody that can reduce the mortality of severe and critical COVID-19 patients. These results should be confirmed in a prospective randomized study.

## Data Availability Statement

Data will be available after article publication and will end 36 months following publication. The information will be shared with researchers whose proposed use of the data has been approved by an independent review committee identified for this purpose.

## Ethics Statement

The studies involving human participants were reviewed and approved by Julio Trigo Hospital and Salvador Allende Hospital. Written informed consent for participation was not required for this study in accordance with the national legislation and the institutional requirements.

## Author Contributions

TC, MR, DS, and PL-L designed the clinical trial and CRFs of the clinical trial. HD, JJ, AH, LV, AM, LPo, RH, NT, and JHJ administered the experimental drug plus the SOC and followed the COVID-19 patients at the hospital intensive care unit. LM, MT, YS, AG, DB, AV, LC, MD, and LPé were responsible of monitoring and data management. PL-L and LS did data processing. DS, MR, and TC interpreted the final results. All authors reviewed and approved the final manuscript.

## Funding

This research was funded by the Cuban Ministry of Health and the Center of Molecular Immunology.

## Conflict of Interest

LM, MT, AG, DB, AV, LC, MD, LPé, PL-L, LS, DS, MR, and TC currently work for the Center of Molecular Immunology, the institution that generated and originally patented nimotuzumab. The rest of the authors do not have any commercial or financial relationships that could be taken as a potential conflict of interest. The remaning authors declare that the research was conducted in the absence of any commercial or financial relationships that could be construed as a potential conflict of interest.

## Publisher's Note

All claims expressed in this article are solely those of the authors and do not necessarily represent those of their affiliated organizations, or those of the publisher, the editors and the reviewers. Any product that may be evaluated in this article, or claim that may be made by its manufacturer, is not guaranteed or endorsed by the publisher.
